# Pharmacologic Activation of TRPA1 Induces Multi-Target Anticancer Responses via Apoptotic and Mitochondrial Pathways

**DOI:** 10.3390/ph19071080

**Published:** 2026-07-13

**Authors:** Murat Çakır, Ali Aydın, Burçin Türkmenoğlu, Mücahit Seçme

**Affiliations:** 1 Department of Physiology, Faculty of Medicine, Yozgat Bozok University, Yozgat 66200, Türkiye; 2 Department of Basic Medical Science, Faculty of Medicine, Yozgat Bozok University, Yozgat 66200, Türkiye; ali.aydin@yobu.edu.tr; 3 Department of Analytical Chemistry, Faculty of Pharmacy, Erzincan Binali Yıldırım University, Erzincan 24002, Türkiye; burcin.turkmenoglu@erzincan.edu.tr; 4 Department of Basic Medical Science, Faculty of Medicine, Ordu University, Ordu 52200, Türkiye; mucahitsecme@odu.edu.tr

**Keywords:** ASP7663, HC030031, anticancer, transient receptor potential channels, Transient Receptor Potential Ankyrin 1, UN SDG 3: Good Health and Well-being

## Abstract

**Objectives**: Transient Receptor Potential Ankyrin 1 (TRPA1) has emerged as a stress-responsive ion channel involved in calcium homeostasis, redox signaling, migration, and cancer cell survival; however, the therapeutic relevance of TRPA1 activation versus inhibition remains poorly understood. In this study, we comparatively investigated the anticancer potential of the TRPA1 agonist ASP7663 and the TRPA1 antagonist HC030031 across a broad panel of human cancer cell lines. **Methods**: Antiproliferative and cytotoxic effects were evaluated using MTT and LDH assays, while apoptotic signaling, DNA fragmentation, mitochondrial membrane potential, migration inhibition, DNA/BSA interactions, topoisomerase I inhibition, and molecular docking analyses were comprehensively assessed. **Results**: ASP7663 exhibited markedly lower GI_50_ values than HC030031 in most cancer models and demonstrated favorable tumor selectivity relative to normal cells. Mechanistically, ASP7663 induced robust apoptotic activation characterized by significant upregulation of Caspase-3, Caspase-8, and Caspase-9, together with enhanced DNA fragmentation and pronounced nuclear condensation. Rhodamine-123 staining further revealed substantial mitochondrial membrane depolarization, indicating activation of intrinsic apoptotic pathways. In addition, ASP7663 produced selective membrane damage in malignant cells, stronger inhibition of migration in osteosarcoma and chondrosarcoma models, enhanced CT-DNA/BSA binding affinity, partial topoisomerase I inhibition, and superior docking interactions with apoptosis-related targets, including Caspase-3, Caspase-8, Caspase-9, Bax, and Bcl-2. **Conclusions**: Collectively, these findings suggest that ASP7663 is a promising multi-target candidate for anticancer therapy and support further investigation of TRPA1-associated pathways as potential therapeutic targets in cancer.

## 1. Introduction

Cancer remains one of the leading causes of death worldwide despite major advances in surgery, radiotherapy, immunotherapy, and targeted therapies [1]. However, treatment resistance, recurrence, metastasis, and systemic toxicity still limit long-term clinical success [2,3]. Therefore, identification of new molecular targets regulating proliferation, apoptosis, and migration remains a major priority in oncology [4].

Ion channels are increasingly recognized as important regulators of tumor biology through their effects on membrane potential, calcium signaling, metabolism, migration, and survival pathways [5,6]. Among them, Transient Receptor Potential Ankyrin 1 is a non-selective Ca^2+^-permeable cation channel activated by oxidative, electrophilic, and inflammatory stress [7,8]. Although first characterized in nociception, TRPA1 is now known to be expressed in epithelial, stromal, and malignant cells [9,10].

Recent studies indicate that TRPA1 has a dual role in cancer. In some tumors, it promotes survival by helping cells tolerate reactive oxygen species (ROS) and microenvironmental stress [11,12,13]. In contrast, excessive pharmacologic activation has been associated with calcium overload, mitochondrial dysfunction, oxidative stress, and apoptotic cell death in experimental models [10,14]. This context-dependent behavior suggests that TRPA1 may function either as a tumor-supportive sensor or as an exploitable anticancer vulnerability.

Calcium signaling is especially relevant because intracellular Ca^2+^ controls cell-cycle progression, mitochondrial energetics, caspase activation, and motility [15,16]. Sustained calcium influx can destabilize mitochondrial membrane potential, trigger cytochrome c release, and activate Caspase-9/Caspase-3-mediated apoptosis [17,18]. In addition, ion-channel signaling contributes to epithelial–mesenchymal transition and metastatic migration through cytoskeletal remodeling [5,6].

ASP7663 is a selective TRPA1 agonist, whereas HC030031 is one of the best-known TRPA1 antagonists used in mechanistic studies [19,20]. Despite their pharmacological relevance, their comparative anticancer potential remains unclear. Because agonism and antagonism may generate fundamentally different stress responses, evaluating both compounds may clarify whether TRPA1 activation or inhibition is more beneficial in tumor cells.

## 2. Results

### 2.1. Evaluation of Cell Proliferation Measurement

The MTT assay results revealed that ASP7663 exerted stronger antiproliferative effects than HC030031 across all tested cancer cell lines. ASP7663 displayed GI_50_ values ranging from 4.28 µM (A549) to 6.12 µM (MCF7), while HC030031 showed slightly higher GI_50_ values between 4.90 µM and 7.68 µM (Table 1). Among lung cancer models, ASP7663 demonstrated particularly strong activity in A549 cells (GI_50_: 4.28 µM), outperforming HC030031 (GI_50_: 4.90 µM) (Table 1). Similar trends were observed in ovarian (A2780, A2780ADR) and colon cancer (HT29, SW620) models (Table 1).

TGI values further supported the superior growth-inhibitory capacity of ASP7663, particularly in rapidly proliferating cells such as A549 (16.94 µM vs. 89.87 µM for HC030031) and HT29 (22.07 µM vs. 413.06 µM) (Table 1). In contrast, HC030031 exhibited substantially higher TGI values across most cell lines, indicating weaker suppression of total cell growth.

Both compounds showed LC_50_ values exceeding the highest tested concentrations (>1899.26 µM for ASP7663 and >1406.87 µM for HC030031), suggesting that neither compound induces strong cytotoxicity at pharmacologically relevant doses (Table 1).

In normal cell lines (FL, HC, Beas-2B), GI_50_ values for ASP7663 (5.08–7.15 µM) were comparable to those observed in cancer cells, while HC030031 showed similar behavior (5.77–7.68 µM), indicating limited selectivity but acceptable safety margins given the absence of cytotoxicity (Table 1).

A key distinguishing parameter was the Tumor Selectivity Index (TSI), calculated from TGI values as the ratio of TGI concentration in normal cells to that in cancer cells. ASP7663 exhibited significantly higher TSI values across most cancer cell lines, with particularly notable selectivity in A549 (TSI: 15.41), A2780 (11.25), HT29 (11.83), and A2780ADR (8.00) (Table 1). In contrast, HC030031 displayed lower TSI values, generally ranging between 0.67 and 7.71, indicating weaker discrimination between cancer and normal cells (Table 1).

### 2.2. Evaluation of Cytotoxic Activity of Test Molecules

The LDH cytotoxicity assay demonstrated that both compounds induced relatively low to moderate membrane damage at their GI50 concentrations across the tested cancer cell lines. ASP7663 exhibited higher cytotoxicity than HC030031 in nearly all cell types. The highest LDH release was observed in A549 lung cancer cells treated with ASP7663 (16.93%), whereas most other cancer cell lines showed cytotoxicity values ranging between approximately 5% and 11% (Table 2). In contrast, HC030031 produced consistently lower LDH release, with values generally remaining below 10% (Table 2).

Among gastrointestinal and bone-related cancer models, ASP7663 caused moderate cytotoxicity in HT29, SW620, MG63, Saos2, and SW1353 cells, while HC030031 showed comparatively weaker membrane-disruptive effects. Importantly, both compounds induced minimal LDH release in normal cell lines, including FL, HC, and Beas2B cells. Particularly, HC030031 produced cytotoxicity values close to 2–2.5% in normal cells, suggesting a favorable selectivity profile (Table 2).

Overall, the relatively low LDH levels indicate that the antiproliferative effects of these compounds are unlikely to result primarily from acute necrotic membrane destruction. Instead, the findings support the possibility that these molecules exert their anticancer activity predominantly through regulated cell death pathways, such as apoptosis-related mechanisms.

### 2.3. Gene Expression Analysis Results of Test Items

Gene expression analysis revealed distinct apoptotic signaling patterns induced by ASP7663 and HC030031. In ASP7663-treated cells, significant upregulation of *Caspase-3* was observed across all cell lines, including A549 (2.77-fold), MG63 (4.20-fold), and notably, Beas-2B (7.41-fold) (Table 3). *Caspase-8* expression was strongly increased in cancer cells, particularly MG63 (6.45-fold) and A549 (3.23-fold), indicating activation of the extrinsic apoptotic pathway. *Caspase-9* showed a mixed response, with upregulation in MG63 (4.81-fold) but downregulation in A549 (−1.51-fold), suggesting cell-type-specific modulation of the intrinsic pathway (Table 3).

Pro-apoptotic *Bax* expression was moderately increased in MG63 (1.32-fold) but downregulated in A549 and Beas-2B. Importantly, ASP7663 caused downregulation of the anti-apoptotic gene *Bcl-2* in A549 (−1.94-fold) and Beas-2B (−1.77-fold), supporting induction of apoptosis through mitochondrial destabilization (Table 3).

HC030031 demonstrated a different profile. In A549 cells, strong upregulation of *Bax* (9.00-fold) and *Caspase-8* (4.30-fold), combined with marked downregulation of *Bcl-2* (−6.19-fold), indicated robust activation of intrinsic apoptosis (Table 3). In MG63 cells, both *Caspase-3* (5.39-fold) and *Caspase-8* (5.19-fold) were significantly increased, suggesting combined pathway activation. However, in normal cells (Beas-2B and HC), HC030031 induced inconsistent or suppressive effects, including strong downregulation of *Caspase-9* (−7.78-fold in Beas-2B) (Table 3).

Overall, ASP7663 exhibited more uniform caspase activation across cell types, whereas HC030031 showed stronger but more variable apoptotic responses.

### 2.4. Determination of DNA Banding Potential of Test Substances

**Figure 1:**
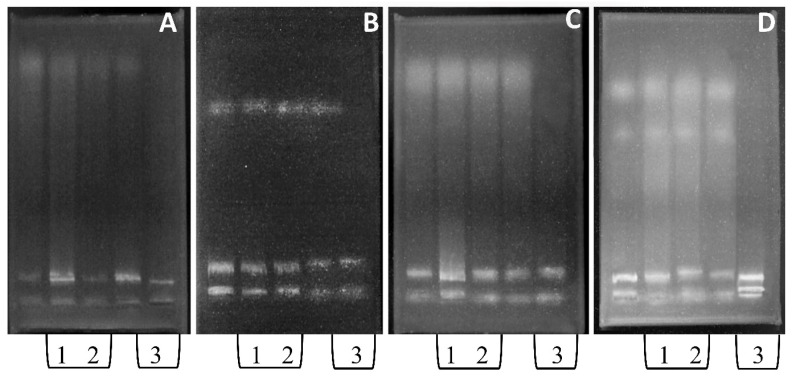
Effect of tested ASP7663 and HC030031 on DNA fragmentation in A549 lung cancer cells (**A**), Beas2B lung normal cells (**B**), MG63 bone cancer cells (**C**), and HC bone normal cells (**D**) (**1.** ASP7663, **2**. HC030031, and **3**. Control).

The DNA banding assay was performed to evaluate the effects of the TRPA1 channel agonist ASP7663 and the TRPA1 channel antagonist HC030031 on DNA degradation, which serves as an indicator of apoptosis in A549 lung and MG63 bone cancer cells, as well as in Beas2B normal lung and HC normal chondrocyte cells (Figure 1).

The results of the DNA banding assay indicated that molecules ASP7663 caused some DNA degradation compared to the control (Figure 1). However, when comparing the cancer and control cells, a lower degree of DNA degradation caused by ASP7663 was observed in the control cells. When HC030031 is compared to ASP7663, it is observed that it does not cause significant DNA damage. Additionally, it is evident from Figure 1 that HC030031 has the least impact on normal cells. These results are consistent with our previous MTT, LDH, and gene expression analyses, confirming that ASP7663 is selectively effective against cancer cells by targeting the apoptotic pathway.

### 2.5. Evaluation of Fluorescent Staining Results of Test Items

**Figure 2:**
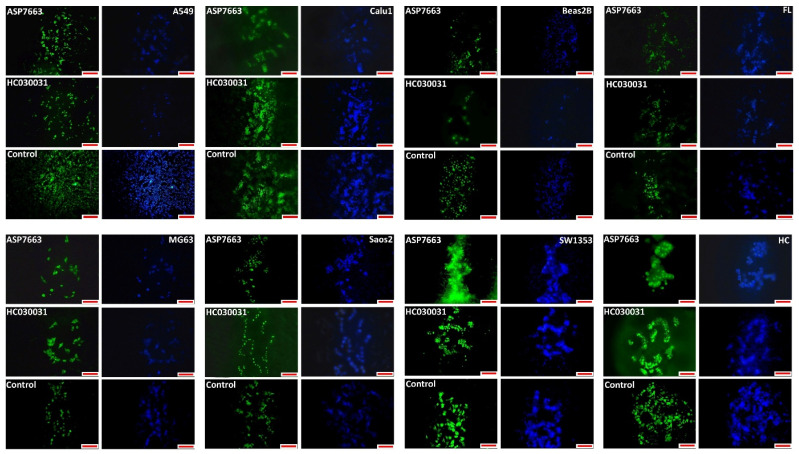
DAPI/Rhodamine 123 staining in A549, Calu1, MG63, Saos2, and SW1353 cancer cell lines and Beas2B and FL normal cell lines. All scales are 100 µm.

To determine the effects of the TRPA1 channel agonist ASP7663 and the TRPA1 channel antagonist HC030031 on mitochondrial membrane potential and cell death, A549, Calu1, MG63, Saos2, and SW1353 cancer cell lines, along with Beas2B, FL, and HC normal cell lines, were stained with DAPI (blue) and Rhodamine123 (green) fluorescent dyes and examined under a fluorescence microscope. DAPI staining indicated that the ASP7663 molecule increased cell death more than the HC030031 (the nucleus was intensely stained blue in the treated cells) (Figure 2).

Rhodamine123 staining also demonstrated that the ASP7663 molecule caused a more significant decrease in mitochondrial membrane potential than the HC030031 (intensity decreased in treated cells, while it remained intensely green in the control) (Figure 2). In summary, an increase in cell death and a decrease in mitochondrial membrane potential were observed after applying the ASP7663 molecule to cancer cells.

### 2.6. Determination of DNA/BSA Binding Constants of Test Items

Many anticancer and other effective drug molecules today sometimes exert their pharmacological effects by binding to DNA biomacromolecules. The transport of these molecules in the blood primarily occurs via the albumin protein (Human Serum Albumin, HSA). Therefore, the DNA/BSA (Bovine Serum Albumin) binding properties of the TRPA1 channel agonist ASP7663 and the TRPA1 channel antagonist HC030031 were determined and are presented here.

**Figure 3:**
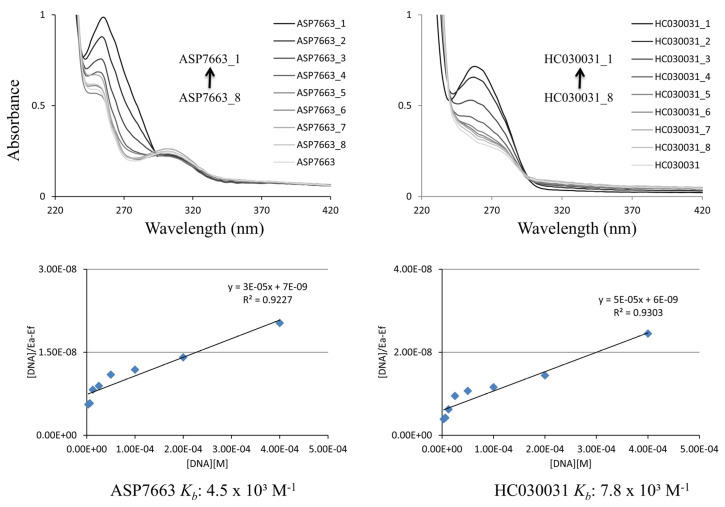
DNA Binding Result. UV/Vis absorption spectrum in the absence and presence of DNA 5 µM (8), 10 µM (7), 20 µM (6), 40 µM (5), 60 µM (4), 80 µM (3), 100 µM (2), and 200 µM (1).

**Figure 4:**
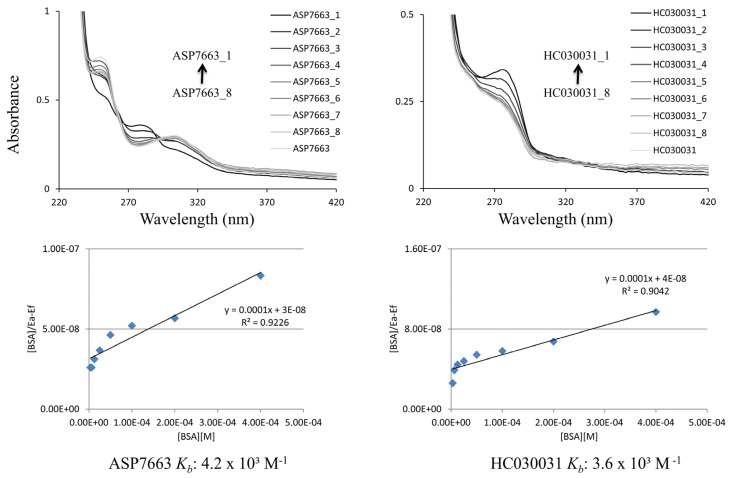
BSA Binding Result. UV/Vis absorption spectrum in the absence and presence of BSA 5 µM (8), 10 µM (7), 20 µM (6), 40 µM (5), 60 µM (4), 80 µM (3), 100 µM (2), and 200 µM (1).

Figure 3 shows the interaction graphs of the test molecules with CT-DNA (Calf thymus DNA), while Figure 4 presents the interaction graphs of the test molecules with BSA. Accordingly, the binding constants (K*_b_*) of ASP7663 and HC030031 with CT-DNA were calculated to be 4.5 × 10^3^ and 7.8 × 10^3^ M^−1^, respectively (Figure 3). Similarly, the binding constants (K*_b_*) of ASP7663 and HC030031 with BSA were calculated as 4.2 × 10^3^ and 3.6 × 10^3^ M^−1^, respectively (Figure 4). Figure 3 and Figure 4 show a maximum peak at 258 nm for the ASP7663-DNA and 278 nm for the ASP7663-BSA interaction. The maximum peak was recorded at 258 nm for the HC030031-DNA interaction in Figure 3, while the maximum peak was observed at 276 nm for the HC030031-BSA interaction in Figure 4. The absorbance increases as the CT-DNA/BSA concentration rises. This phenomenon, called the hyperchromic effect, suggests that the test substances interact with CT-DNA/BSA. This interaction likely occurs through the test substance/CT-DNA intercalation mode. Additionally, these changes in the absorption spectrum indicate that at least part of the interaction may occur through binding to DNA cavities.

### 2.7. Effect of Test Substances on Topoisomerase I

**Figure 5:**
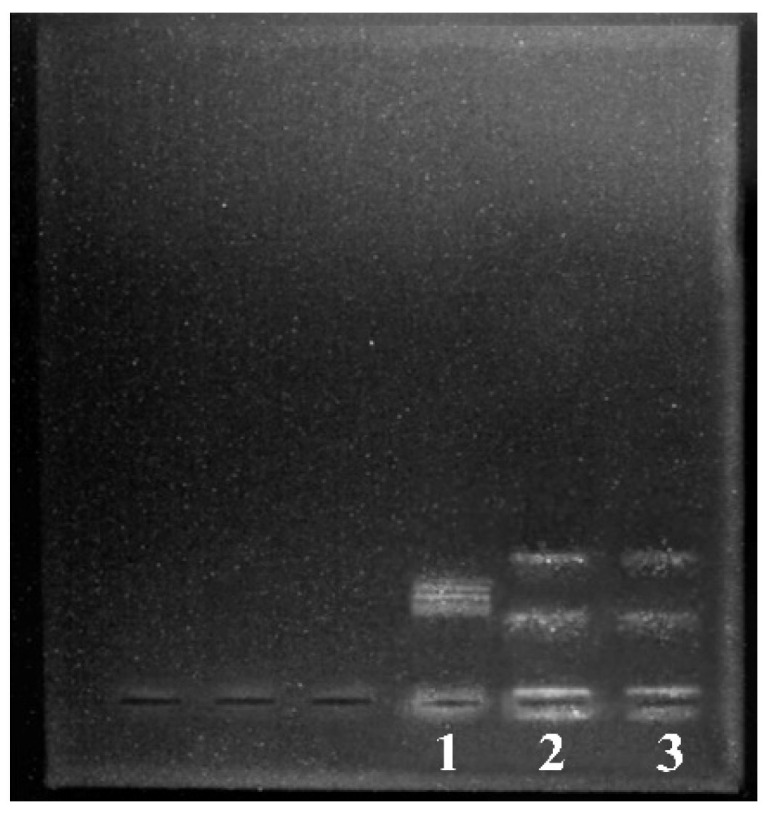
Effect of test substances on topoisomerase I. Control (1), HC030031 (2), and ASP7663 (3).

In this study, the effects of the tested TRPA1 channel agonist, ASP7663, and the TRPA1 channel antagonist, HC030031, on topoisomerase I activity were determined using a topoisomerase I inhibition test. When comparing the test results to the control, it is evident from Figure 5 that the molecules ASP7663 and HC030031 partially inhibited the DNA relaxing activity of topoisomerase I at TGI concentrations. Based on these results, it can be concluded that the inhibition of topoisomerase I contributes to some of the anticancer effects of the tested substances.

### 2.8. Effect of Test Substances on Cell Migration

The migration capacity of cells is an essential characteristic of cancer and a target for new anticancer agents. Cancer cells with migration capacity can evade the apoptosis mechanism. Therefore, one of the goals of newly developed anticancer drugs is to reduce cancer cells’ migration capacity significantly. According to the time-dependent migration assay, the test substances significantly suppressed the migration capacity of A549, Calu1, Saos2, MG63, and SW1353 cells compared to control cells (Supplemental Figures S1 and S2). Compared to the control, these substances administered at the GI50 concentration significantly reduced the migration rate in cell lines. Because Day 3 values correspond to the remaining open gap, higher percentages reflect stronger suppression of cell migration. In untreated controls, Day 3 gaps were 0% in nearly all cell lines, indicating complete wound closure and normal migratory behavior. ASP7663 significantly inhibited migration in multiple models. In A549 cells, a 17.46% gap remained on Day 3, while in Calu1 cells, 14.52% remained (Table 4). More pronounced inhibition was observed in mesenchymal-related and normal stromal models, including FL fibroblasts (54.01%), HC osteoblast-like cells (54.63%), Saos2 (53.57%), and MG63 (63.17%). SW1353 cells also retained a substantial residual gap (44.09%) (Table 4). HC030031 demonstrated even stronger inhibition in several cell lines. In A549 and Calu1 cells, Day 3 residual gaps remained at 55.87% and 50.85%, respectively, indicating markedly greater migration suppression than ASP7663 (Table 4). In Saos2 cells, HC030031 produced a 36.98% remaining gap, while SW1353 cells showed 27.35%. In MG63 cells, strong inhibition was also observed (61.02%) (Table 4). HC osteoblast-like normal cells retained a 53.16% gap, and FL fibroblasts showed the highest residual gap value at 65.14%, indicating profound suppression of motility (Table 4). Overall, both compounds significantly inhibited migration compared with controls, with HC030031 producing stronger effects in A549, Calu1, and FL cells, while ASP7663 was comparatively stronger in Saos2 and SW1353. Since scratch closure depends on both migration and proliferation, these results should be interpreted alongside prior MTT data showing antiproliferative effects. Therefore, the observed inhibition likely reflects a combined suppression of locomotion and growth, which is desirable in anti-metastatic therapy. Overall, HC030031 may be stronger in certain epithelial/stromal models, whereas ASP7663 demonstrates potent and consistent inhibition in bone-associated cancer models.

### 2.9. Molecular Docking Results

ASP7663 and HC030031 were evaluated using molecular docking, a structure-based computational approach employed to predict potential interactions between small molecules and protein targets. Caspase-3 (5IAG), Caspase-8 (1QTN), Caspase-9 (2AR9), Bax (1F16), and Bcl-2 (4IEH) were selected as apoptosis-related targets based on the experimental findings. The predicted binding affinities and interaction parameters obtained for ASP7663 and HC030031 are summarized in Table 5.

Table 5 shows the compounds (ASP7663 and HC030031) docking score, Glide emodel, Glide energy (MM/GBSA ΔG_Bind_), MM/GBSA ΔG_Bind_ Coulomb, and MM/GBSA ΔG_Bind_ Covalent values, which were calculated and determined. These values reveal the effectiveness of the energy between the ligand and the target. The docking results suggested that ASP7663 generally exhibited more favorable predicted binding interactions with the investigated targets than HC030031.

Caspase 3, which is important in the apoptosis pathway in vitro experimental studies, was identified as the first target. The binding values of compounds ASP7663 and HC030031, which interact with the crystal structure of Caspase 3, are presented in Table 5. The docking scores of the interactions of compounds ASP7663 and HC030031 with Caspase-3 are −6.915 and −4.931 kcal/mol, respectively. ASP7663 exhibited the most favorable docking score against Caspase-3 5IAG. The Glide emodel value was determined as −34.832, the Glide energy as −27.100 kcal/mol, and the ΔG_Bind_ free binding energy values were determined as −32.39 kcal/mol. Visual representations of the interactions between Caspase-3 and ASP7663 or HC030031 are presented in Supplemental Figures S3 and S4. Supplemental Figure S3 shows the 2D and 3D interaction diagrams of the best-interacting ASP7663. Supplemental Figure S3 shows that Asp7663, which is docked in the active pocket region, interacts partially hydrophobically via -COO-, through hydrogen bonds with amino acid residues Arg64 and Gln161, and Cys163, and through a salt bridge with amino acid residue Arg207.

Caspase 8, another important target in the cancer signaling pathway, was investigated by molecular docking. Binding values (Table 5) and binding modes (Supplemental Figures S5 and S6) on Caspase 8 were determined for both compounds. The docking scores for the interactions of Caspase 8 with ASP7663 and HC030031 were −4.204 and −4.088 kcal/mol. ASP7663 had the best interaction with this target, followed by HC030031. The ΔG_Bind_ energy of the ligand/target complex formed by Caspase 8/ASP7663 in molecular docking was −27.54 kcal/mol. ASP7663, docked in the main groove of the Caspase 8 crystal structure, has hydrogen bond interactions with Arg413 and Gln358 amino acids via the -COO- group, salt bridge interactions with the amino acid residue Arg260, and hydrogen bond interactions with Arg413 via the =O atom, determined according to 2D and 3D diagrams (Supplemental Figure S5).

Caspase 9 is a well-known target, crucial for apoptosis. Therefore, it was identified as another target for in silico approaches. Binding values for compounds ASP7663 and HC030031 that interact with Caspase 9 are presented in Table 5. Based on these data, ASP7663 was theoretically the most likely compound to interact. The docking score, Glide emodel, Glide energy, ΔG_Bind_, ΔG_Bind_ Columb, and ΔG_Bind_ covalent values for the interaction between Caspase 9 and ASP7663 in Table 5 are −6.751, −32.101, −29.567, −29.97, −71.33, and 0.02 kcal/mol, respectively. In addition, in the 2-dimensional diagram obtained for Caspase 9 interacting with ASP7663 in molecular docking, there is a hydrogen bond interaction between the amino acids Arg180 and Gln285 via the -COO- group and the amino acids Ser287 and Ser353 via the =O (Supplemental Figure S7). Supplemental Figure S8 shows the crystal structure of Caspase 9 with HC030031.

Because the increase in B cell lymphoma 2 (Bcl-2) and Bax expression is important in tumorigenesis and anticancer treatment, they were identified as other targets investigated in molecular docking studies. Table 5 presents the values for two compounds interacting with the crystal structure of Bcl-2 obtained from the protein data bank. The docking scores of Bcl-2 calculated for ASP7663, HC030031, are −4.764 and −4.083 kcal/mol, respectively. Table 5 shows that the binding values for Bcl-2 for both compounds are quite close to each other. The free binding energy values, known as the numerical value of the target/ligand complex formed in molecular docking, are −32.15 and −47.62 kcal/mol for ASP7663 and HC030031, respectively. In Supplemental Figures S9 and S10, ASP7663 is docked in the active pocket region of the 3D crystal structure of Bcl-2, and in the 2D diagram, it has hydrogen bonds and hydrophobic interactions with the amino acid residues Tyr67 and Arg66 via the -COO- group.

As mentioned, Bax expression is very important in anticancer studies. Therefore, Bax was chosen as the final target for in silico approaches. ASP7663 and HC030031 were interacted in silico with the crystal structure of the Bax target, and binding parameter values were obtained (Table 5). ASP7663 had the best binding value, similar to the docking scores obtained from the other targets. The docking scores of ASP7663 and HC030031 interacting with the Bax target were −4.028 and −2.851 kcal/mol, respectively. ASP7663 showed the most favorable predicted interaction with Bax, with Glide emodel, Glide energy, and MM/GBSA ΔGBind values of −26.400, −23.991, and −29.56 kcal/mol, respectively. 2D diagrams of these interactions are presented in Supplemental Figures S11 and S12. The 2D and 3D complex structures of the molecular docking interaction of ASP7663 with the crystal structure of Bax are presented in Supplemental Figure S11. It was determined that ASP7663, docked in the active pocket region of the crystal structure, exhibits π-π stacking interactions with the amino acid Trp206 via the phenyl ring and hydrogen bonding interactions with the amino acid Asn208 via the -N atom.

## 3. Discussion

TRPA1 has increasingly emerged as a relevant regulator in cancer biology. Initially associated with nociception, inflammation, and irritant sensing, TRPA1 is now linked to tumor proliferation, migration, oxidative stress adaptation, apoptosis, and therapy resistance. As a redox-sensitive and Ca^2+^-permeable channel, TRPA1 integrates calcium remodeling with cellular stress signaling [14,26,27,28]. In the present study, ASP7663 exerted stronger antiproliferative, cytotoxic, pro-apoptotic, and anti-migratory effects than HC030031 across multiple cancer cell models. These findings suggest that pharmacological modulation of TRPA1-associated pathways may represent a promising therapeutic strategy and warrant further mechanistic investigation.

The biological consequences of TRPA1 activation appear highly dependent on the amplitude and duration of Ca^2+^ influx. Oscillatory Ca^2+^ signals can support proliferation and survival through MAPK/ERK, PI3K/AKT, and transcriptional programs, whereas sustained Ca^2+^ overload promotes mitochondrial depolarization, cytochrome c release, and apoptotic death [15,29,30,31]. The observed activation of Caspase-3, Caspase-8, and Caspase-9 together with DNA fragmentation indicates that ASP7663 may simultaneously engage both intrinsic and extrinsic apoptotic pathways. This observation is consistent with previous reports suggesting that dysregulated calcium signaling between mitochondria and the endoplasmic reticulum can sensitize cancer cells to apoptotic signaling [32,33,34].

An important implication of these findings is that TRPA1 may function as a regulator of cellular stress responses in tumors. Cancer cells frequently experience elevated oxidative stress and rely on adaptive signaling pathways to maintain survival [35,36]. Previous studies have suggested that TRPA1 contributes to cellular adaptation to oxidative and metabolic stress through its role as a redox-sensitive ion channel [11,14,27]. Excessive pharmacological modulation of TRPA1 may disrupt these adaptive processes, potentially promoting mitochondrial dysfunction and apoptotic signaling [14,29,31]. However, because intracellular ROS levels were not directly measured in the present study, the precise contribution of oxidative stress to the observed effects remains uncertain and warrants further investigation.

The potential involvement of oxidative stress in ASP7663-induced apoptosis should therefore be interpreted with caution. Although TRPA1 is recognized as a redox-sensitive ion channel and previous reports have linked TRPA1 modulation to ROS-associated cell death pathways, oxidative stress was not directly assessed in the present study. Future studies employing ROS-sensitive fluorescent probes and antioxidant rescue approaches, such as N-acetylcysteine pretreatment, will be necessary to clarify whether redox signaling contributes to the anticancer effects observed following ASP7663 treatment.

An important observation of the present study is the apparent discrepancy between the low GI50 values and the very high LC50 values obtained for both compounds. This finding suggests that ASP7663 and HC030031 primarily exert cytostatic and apoptosis-promoting effects at pharmacologically relevant concentrations rather than inducing rapid necrotic cell death. Consistent with this interpretation, LDH release remained relatively modest, whereas caspase activation, DNA fragmentation, nuclear condensation, and mitochondrial depolarization were clearly observed. Therefore, growth inhibition and apoptotic signaling appear to precede overt cytotoxicity in these cellular models, explaining why substantial biological effects were detected despite LC50 values exceeding the tested concentration range.

The anti-migratory effect of ASP7663 is also translationally significant. Metastasis remains the leading cause of cancer-related mortality, and ion channels are increasingly recognized as regulators of invasion, migration, and metastatic dissemination [5,37]. Ca^2+^ signaling coordinates focal adhesion turnover, cytoskeletal remodeling, and directional motility. Thus, the marked suppression of wound closure observed after ASP7663 treatment suggests disruption of coordinated calcium gradients required for cell movement. This positions ASP7663 not only as a cytotoxic candidate but also as a potential anti-metastatic agent.

Although HC-030031 showed weaker overall efficacy, antagonist strategies may still be advantageous in selected tumors. In cancers where endogenous TRPA1 mainly supports ROS defense, NRF2-linked adaptation, or chemoresistance, TRPA1 blockade may suppress survival signaling and restore treatment sensitivity [11,38,39]. Therefore, agonist-versus-antagonist selection should likely depend on tumor lineage, TRPA1 expression level, oxidative phenotype, and mitochondrial reserve capacity.

Another noteworthy aspect is the possibility of polypharmacology. If ASP7663 also modulates topoisomerase-I activity or apoptosis-related proteins, its superiority may not rely exclusively on TRPA1. Multi-target agents are often advantageous because malignant cells rapidly compensate for single-node inhibition. Concurrent induction of calcium stress, mitochondrial dysfunction, and DNA damage may therefore produce deeper and more durable responses [2,36].

These findings also support combination strategies. Temozolomide resistance in glioblastoma remains a major clinical challenge driven by DNA repair, survival signaling, and adaptive stress pathways [40,41]. Combining TRPA1 agonism with ROS-generating or DNA-damaging therapies may lower the apoptotic threshold. Similarly, co-targeting mitochondrial Ca^2+^ homeostasis and autophagy has been shown to enhance cancer cell chemosensitivity, reinforcing the rationale for calcium-centered combination therapy [42].

Overall, the present data support TRPA1 as a druggable signaling hub in cancer. ASP7663 appears to convert TRPA1 signaling from an adaptive survival pathway into a pro-apoptotic and anti-migratory stress program. Future studies should validate TRPA1 dependency using genetic silencing, real-time Ca^2+^ imaging, ROS flux assays, mitochondrial membrane potential analyses, and in vivo tumor models. Biomarker-guided stratification based on TRPA1 expression, redox status, and mitochondrial reserve may ultimately position TRPA1 modulation within precision oncology.

Several limitations of the present study should be acknowledged. Although ASP7663 and HC030031 are widely used pharmacological modulators of TRPA1, direct confirmation of TRPA1 channel activation or inhibition was not performed. Therefore, the observed antiproliferative, pro-apoptotic, and anti-migratory effects should be interpreted as being associated with pharmacological modulation of TRPA1-related pathways rather than definitive proof of TRPA1-dependent signaling. Future studies employing calcium imaging, electrophysiological recordings, and genetic approaches such as siRNA-mediated knockdown or CRISPR/Cas9-mediated TRPA1 deletion will be necessary to establish the precise contribution of TRPA1 to the observed anticancer responses.

## 4. Materials and Methods

### 4.1. Cancer Cell Lines and Cell Culture

A549 (ATCC, CCL-185), Calu1 (ATCC, HTB-54), and H1650 (ATCC, CRL-5883) lung cancer cell lines, MG63 (ATCC, CRL-1427), SW1353 (ATCC, HTB-94), and Saos2 (ATCC, HTB-85) bone cancer cell lines, HeLa (ATCC, CCL-2), A2780 (RRID, CVCL-0134), and A2780ADR (RRID CVCL-1941) gynecological cancer cell lines, SW620 (ATCC, CCL-227) and HT29 (ATCC, HTB-38) colon cancer cell lines, MCF7 (ATCC, HTB-22) breast cancer cell line, as well as the normal lung cell line Beas2B (RRID, CVCL-0168), normal chondrocyte cell line HC (Sigma Aldrich, 402-05A, Darmstadt, Germany), and normal epithelial cell line FL (ATCC CCL-62), were utilized. The cultured cells were incubated in supplemented DMEM (RPMI 1640 for H1650) medium at 37 °C in 5% CO_2_. When the cell viability rate was 90% or higher, experimental studies commenced, and 7500 cells were seeded into each well of 96-well plates.

### 4.2. Determination of the Effect on Cell Proliferation

The MTT test was used to measure the effects of ASP7663 and HC030031 compounds on cell proliferation [43]. The ASP7663 and HC030031 compounds and the control molecule were tested at concentrations of 0.01, 0.1, 1, 10, and 100 μM. Twenty-four hours after the test molecules were added, a second stock was prepared by diluting 1/9 of a 5 mg/mL MTT stock solution with medium for the MTT test. The medium from the incubated plate was removed, and this MTT solution was added instead. After incubating at 37 °C with 5% CO_2_ for 4 h, absorbance values were measured at 570 and 630 nm. Using these absorbance values, the GI50 value (Growth Inhibition 50%), the TGI value (Total Growth Inhibition), and the LC50 value (Lethal Concentration 50%) were calculated using an Excel program.

### 4.3. Determination of the Effect on the Cell Cytotoxicity

The cell cytotoxic activities of compounds ASP7663 and HC030031 were determined using the LDH method [43]. Measuring the cytoplasmic enzymes released into the medium due to cell damage forms the basis of these experiments. LDH is a stable cytoplasmic enzyme found in most cells. For this purpose, the LDH cell cytotoxicity kit was utilized to measure LDH levels in the medium according to the manufacturer’s instructions. At the end of this process, % cytotoxicity was calculated from the absorbance values obtained by reading at 492–630 nm using the Excel program.

### 4.4. Determination of DNA/BSA Binding Constant

The UV/Vis absorption method was employed for the DNA/BSA binding assay [44]. Experiments testing the interaction of CT-DNA/BSA molecules were conducted in a buffer solution containing 5 mM Tris and 50 mM NaCl, with the pH adjusted to 7.3 using HCl. Stock solutions of the test molecules were prepared at a concentration of 1 × 10^−3^ M in DMSO. Absorption titration experiments were conducted to maintain a constant ligand concentration while gradually increasing the concentration of CT-DNA/BSA. Based on the results obtained, the [DNA/BSA]/(εa − εf) values were plotted against the corresponding DNA/BSA concentrations ([DNA/BSA]) using the Wolfe–Shimmer equation. The slope of the line obtained from the graph yields 1/(εb − εf), while the intercept point provides 1/Kb(εb − εf). The binding constants, Kb, were calculated by dividing the two values by each other. In this equation, εf represents the molar absorption coefficient of the free compound, εb denotes the molar absorption coefficient of the fully bound compound, and εa is A/[M]. Wolfe–Shimmer equation: [DNA/BSA]/(|εA − εF|) = [DNA/BSA]/(|εB − εF|) + 1/{Kb (|εB − εF|)}.

### 4.5. Determination of the Effect on the Expression of Apoptotic Genes

To elucidate the molecular mechanism underlying the anticancer activity of the test substances, we assessed the expression profiles of five apoptosis-related genes (*Caspase-3*, *Caspase-8*, *Caspase-9*, *Bax*, and *Bcl-2*) in cancer cell lines treated with these substances [45]. The *GAPDH* gene was used as a housekeeping gene in normalization studies of the results.

### 4.6. DNA Laddering Test

A DNA banding test was conducted to assess the impact of ASP7663 and HC030031 compounds on DNA [46]. Cultured cells were incubated with the medium for 24 h by pipetting 750.000 cells into each T25 flask. At the end of the incubation period, the cells were detached from the flask using a scraper, washed with DPBS, and transferred to a 15 mL tube. The tubes contained 5 mL of 70% alcohol and were stored at −20 °C for 24 h. The alcohol was discarded, and the samples were dried. Phosphate-citrate buffer was added and kept at 37 °C for 30 min. Next, the samples were centrifuged, and the supernatants transferred to Eppendorf tubes (ISOLAB Laborgeräte GmbH, Eschau, Germany). To this, 5 μL of Tween 20 and RNase were added, then the mixture was incubated at 37 °C for 30 min. After incubation, 5 μL of proteinase K and SDS were added and maintained at 37 °C for 30 min. A 1.5% agarose gel with 5% ethidium bromide was prepared, and the sample DNA with a positive control was loaded onto the gel with loading buffer and run. DNA degradation and banding were visualized using a gel imaging system.

### 4.7. Topoisomerase I Inhibition Test

Topoisomerases are abundant enzymes crucial for all living organisms. DNA topoisomerases act as therapeutic targets for anticancer drugs. Therefore, topoisomerase I activity was measured to understand the effects of the test substances. For this purpose, the Topogen I kit (TopoGEN Inc., Columbus, OH, USA) was used according to the manufacturer’s procedure [44].

### 4.8. Determination of the Effect on the Cell Migration

A wound-healing assay was used to determine the effects of the ASP7663 and HC030031 compounds on cell migration [44]. A “scratch” was created by scraping the cell monolayer in a straight line with a p200 pipette tip (ISOLAB Laborgeräte GmbH, Eschau, Germany). Cell debris was removed, and the cells were washed once with 1 mL of growth medium to smooth the edge of the scratch. Fresh medium was then added for in vitro scratch analysis. Approximately 2 mL of fresh medium was added, and test molecules were placed at the GI50 dose. From this moment, the specimens began to be photographed. Photographs were taken of the marked scratch, where the test substances were applied every 24 h until the gap of approximately 500 μm in the control scratch was filled.

### 4.9. DAPI-Rodamin123 Staining

For DAPI-Rhodamin123, cells were washed with DPBS and incubated with Rhodamin123 dye at 37 °C for 15 min [46]. After this, the cells were washed with DPBS and fixed in cold methanol for 10 min. Subsequently, the cells were washed with DPBS and incubated with DAPI stain at 37 °C for 15 min. Rhodamine123 stain was visualized at 520 nm, while DAPI stain was observed at 490 nm under fluorescence microscopy (Leica Microsystems, Wetzlar, Germany).

### 4.10. Bioinformatics-Supported Molecular Docking Analyses

Minimization of designed compounds, completion of missing hydrogens, and optimization processes in the appropriate pH range were prepared with the LigPrep wizard of the Schrödinger Maestro 14.1 program (Schrödinger, Release 2024) [47]. These prepared compounds were optimized for interaction with the target using the molecular docking method [48,49,50]. ASP7663 and HC030031 compounds were optimized using the LigPrep wizard in the first step and then required to interact with the identified targets. Caspase 3 (5IAG), Caspase 8 (1QTN), Caspase 9 (2AR9), Bax (1F16), and Bcl-2 (4IEH) were identified as targets. According to the experimental study, the crystal structures of the identified targets were obtained from the protein data bank (https://www.rcsb.org/, accessed on 12 February 2026). As stated in previous studies, the crystal structures were prepared using the ProteinPrep wizard in Schrödinger Maestro 14.1. Since the active binding site of each target is different, the binding sites of the crystal structures were determined using the “Receptor Grid Generation” module of Schrödinger Maestro 14.1. The compounds obtained were prepared using LigPrep, and then the binding mechanism was revealed by docking them in the active site region of the target’s crystal structure. All of these calculations were performed using Schrödinger Maestro 14.1. The free binding energies of the complex structure formed between the ligand and the target were also calculated using the MM-GBSA method.

### 4.11. Statistical Analysis

The conformity of the groups to a normal distribution was evaluated using the Kolmogorov–Smirnov test. A one-way analysis of variance was used to compare the groups. Levene’s test was conducted to assess the homogeneity of variances. When the variances were found to be non-homogeneous after the one-way analysis of variance, the Tamhane T2 test was used for multiple comparisons.

## 5. Conclusions

In conclusion, the present study demonstrates that pharmacological modulation of Transient Receptor Potential Ankyrin 1 produces significant anticancer responses, with the TRPA1 agonist ASP7663 consistently outperforming the antagonist HC030031 across multiple experimental platforms. ASP7663 showed superior antiproliferative activity, favorable tumor selectivity, broader activation of apoptotic pathways, stronger DNA fragmentation, marked mitochondrial membrane depolarization, and substantial inhibition of cell migration. In addition, its measurable DNA/BSA binding properties, partial topoisomerase I inhibition, and stronger docking affinity toward apoptosis-related proteins support a multi-target mechanism of action.

These findings suggest that controlled TRPA1 activation can convert a redox-sensitive survival channel into a therapeutic vulnerability, particularly in tumors dependent on oxidative stress adaptation and calcium homeostasis. In contrast, HC030031 displayed more selective and context-dependent effects, especially in migration assays, but lacked the broad-spectrum potency observed with ASP7663.

From a translational perspective, ASP7663 represents a promising lead compound for further anticancer drug development. Its ability to simultaneously suppress proliferation, induce programmed cell death, impair mitochondrial function, and reduce metastatic behavior may offer advantages over single-pathway agents that are prone to resistance. Future investigations should focus on validating TRPA1 dependency using genetic approaches, defining ROS/Ca^2+^ signaling dynamics, optimizing pharmacokinetics, and evaluating in vivo efficacy alone or in combination with cisplatin, paclitaxel, or temozolomide.

Overall, this work identifies TRPA1 agonism as a novel and underexplored therapeutic strategy in oncology and positions ASP7663 as a strong candidate for next-generation multi-target cancer therapy.

## Figures and Tables

**Table 1:** GI50, TGI, and LC50 values of molecules against cell lines.

µM	ASP7663			TSI	HC030031			TSI
	GI50	TGI	LC50		GI50	TGI	LC50	
A549	4.28	16.94	>1899.26	15.41	4.90	89.87	>1406.87	3.23
Calu1	5.26	76.58	>1899.26	3.41	5.70	237.31	>1406.87	1.22
H1650	5.21	142.29	>1899.26	1.83	5.85	217.08	>1406.87	1.33
A2780	4.90	23.21	>1899.26	11.25	5.20	43.73	>1406.87	6.64
A2780ADR	5.18	32.63	>1899.26	8.00	5.39	37.66	>1406.87	7.71
HeLa	5.46	197.83	>1899.26	1.32	6.61	224.14	>1406.87	1.29
HT29	5.93	22.07	>1899.26	11.83	6.19	413.06	>1406.87	0.70
SW620	5.43	45.09	>1899.26	5.79	5.77	431.65	>1406.87	0.67
MCF7	6.12	101.80	>1899.26	2.56	6.33	290.43	>1406.87	1.00
Saos2	6.00	401.67	>1899.26	0.65	6.27	434.95	>1406.87	0.66
SW1353	5.20	276.12	>1899.26	0.94	6.36	303.18	>1406.87	0.95
MG63	5.29	264.28	>1899.26	0.98	5.94	294.40	>1406.87	0.98
FL	5.08	256.00	>1899.26		5.77	292.94	>1406.87	
HC	5.93	304.42	>1899.26		6.70	310.07	>1406.87	
Beas2B	7.15	223.13	>1899.26		7.68	268.91	>1406.87	

**Table 2:** % Cytotoxicity values of test substances at GI50 concentrations.

**% Cytotoxicity**	**A549**	**Calu1**	**H1650**	**A2780**	**A2780ADR**	**HeLa**	**MCF7**	
ASP7663	16.93	8.93	10.89	11.45	10.28	6.32	7.58	
HC030031	9.26	5.43	3.59	8.17	12.89	4.95	4.92	
**% Cytotoxicity**	**HT29**	**SW620**	**MG63**	**Saos2**	**SW1353**	**FL**	**HC**	**Beas2B**
ASP7663	11.11	9.63	5.36	6.12	5.05	4.99	3.50	4.49
HC030031	3.38	4.12	3.40	4.61	3.69	2.55	2.44	2.23

**Table 3:** Expression levels of genes in cells treated with ASP7663 (**A**) and HC030031 (**B**).

**A**	**A549**		**Beas2B**		**MG63**		**HC**	
**Gene**	FR *	***p*** **-Value**	**FR**	***p*** **-Value**	**FR**	***p*** **-Value**	**FR**	***p*** **-Value**
*GAPDH*	1.00	non	1.00	non	1.00	non	1.00	non
*Caspase-3*	2.77	0.034	7.41	0.002	4.20	0.040	3.48	0.041
*Caspase-8*	3.23	0.016	1.69	0.014	6.45	0.000	−2.00	0.043
*Caspase-9*	−1.51	0.038	3.57	0.015	4.81	0.003	−3.81	0.008
*Bax*	−1.05	0.041	−1.72	0.012	1.32	0.034	1.05	0.037
*Bcl-2*	−1.94	0.025	−1.77	0.022	2.59	0.004	1.19	0.046
**B**	**A549**		**Beas2B**		**MG63**		**HC**	
**Gene**	**FR**	***p*** **-Value**	**FR**	***p*** **-Value**	**FR**	***p*** **-Value**	**FR**	***p*** **-Value**
*GAPDH*	1.00	non	1.00	non	1.00	non	1.00	non
*Caspase-3*	1.63	0.018	−1.24	0.039	5.39	0.048	1.12	0.038
*Caspase-8*	4.30	0.000	1.03	0.043	5.19	0.000	−2.47	0.032
*Caspase-9*	1.28	0.045	−7.78	0.000	2.08	0.032	−1.61	0.046
*Bax*	9.00	0.037	−3.36	0.002	1.86	0.002	1.05	0.036
*Bcl-2*	−6.19	0.015	−1.33	0.021	1.17	0.045	1.02	0.021

* Fold Regulation.

**Table 4:** The migration analysis of the cells with ImageJ (version 1.54).

**(% Area)**	**A549**		**Calu1**		**Beas2B**		**FL**	
	**Gap** **Day 1**	**Gap** **Day 3**	**Gap** **Day 1**	**Gap** **Day 3**	**Gap** **Day 1**	**Gap** **Day 3**	**Gap** **Day 1**	**Gap** **Day 3**
ASP7663	74.13	17.46	51.92	14.52	50.77	13.15	60.30	54.01
HC030031	62.87	55.87	46.09	50.85	46.74	44.30	47.66	65.14
Control	50.28	0.00	48.21	0.00	50.90	0.00	31.93	0.00
**(% Area)**	**Saos2**		**MG63**		**SW1353**		**HC**	
	**Gap** **Day 1**	**Gap** **Day 3**	**Gap** **Day 1**	**Gap** **Day 3**	**Gap** **Day 1**	**Gap** **Day 3**	**Gap** **Day 1**	**Gap** **Day 3**
ASP7663	55.41	53.57	70.91	63.17	59.37	44.09	71.54	54.63
HC030031	55.54	36.98	62.33	61.02	69.77	27.35	50.03	53.16
Control	42.25	0.00	42.27	0.00	53.02	0.00	35.96	0.00

**Table 5:** Calculated values of compounds interacting with the crystal structure of 6JQR.

Target	Crystal Structure	Binding Values (kcal/mol)	ASP7663	HC030031
Caspase 3	5IAG [21]	Docking score	−6.915	−4.931
		Glide emodel	−34.832	−47.575
		Glide energy	−27.100	−37.211
		MM/GBSA ΔG_Bind_	−32.39	−49.49
		MM/GBSA ΔG_Bind_ Coulomb	−37.29	4.45
		MM/GBSA ΔG_Bind_ Covalent	1.94	−0.60
Caspase 8	1QTN [22]	Docking score	−4.204	−4.088
		Glide emodel	−26.990	−50.229
		Glide energy	−26.671	−38.669
		MM/GBSA ΔG_Bind_	−27.54	−39.27
		MM/GBSA ΔG_Bind_ Coulomb	−9.60	−3.75
		MM/GBSA ΔG_Bind_ Covalent	3.21	1.53
Caspase 9	2AR9 [22,23]	Docking score	−6.751	−3.145
		Glide emodel	−32.101	−41.715
		Glide energy	−29.567	−32.632
		MM/GBSA ΔG_Bind_	−29.97	−39.48
		MM/GBSA ΔG_Bind_ Coulomb	−71.33	−17.26
		MM/GBSA ΔG_Bind_ Covalent	0.02	8.42
Bcl-2	4IEH [24]	Docking score	−4.764	−4.083
		Glide emodel	−28.254	−47.141
		Glide energy	−25.088	−37.141
		MM/GBSA ΔG_Bind_	−32.15	−47.62
		MM/GBSA ΔG_Bind_ Coulomb	−3.63	−6.55
		MM/GBSA ΔG_Bind_ Covalent	1.52	3.01
Bax	1F16 [25]	Docking score	−4.028	−2.851
		Glide emodel	−26.400	−42.771
		Glide energy	−23.991	−35.959
		MM/GBSA ΔG_Bind_	−29.56	−40.74
		MM/GBSA ΔG_Bind_ Coulomb	−28.34	−13.41
		MM/GBSA ΔG_Bind_ Covalent	2.01	1.92

## Data Availability

The datasets used and analyzed during the current study are available from the corresponding author upon reasonable request. All data analyzed during this study are included in this published article as tables and figures.

## Supplementary Materials

The following supporting information can be downloaded at https://www.mdpi.com/article/10.3390/ph19071080/s1. Figure S1: Effects of ASP7663 and HC030031 molecules on cell migration in A549, Calu1, Beas2B, and FL cell lines. Tests were performed using cell lines showing exponential growth in log phase, and the incubation period was limited to 3 days. Figure S2: Effects of ASP7663 and HC030031 molecules on cell migration in HC, Saos2, MG63, and SW1353 cell lines. Tests were performed using cell lines showing exponential growth in log phase, and the incubation period was limited to 3 days. Figure S3: 2D and 3D diagrams of the interactions of compound ASP7663 with the crystal structure of Caspase-3 (5IAG). Figure S4: 2D diagrams of the interactions of HC030031 with Caspase 3 (5IAG). Figure S5: 2D and 3D diagrams of the interactions of compound ASP7663 with the crystal structure of Caspase 8 (1QTN). Figure S6: 2D diagram of the interaction of HC030031 with Caspase-8 (1QTN). Figure S7: 2D and 3D diagrams of the interactions of compound ASP7663 with the crystal structure of Caspase 9 (2AR9). Figure S8: 2D diagram of the interaction of HC030031 with Caspase-9 (2AR9). Figure S9: 2D and 3D diagrams of the interactions of compound ASP7663 with the crystal structure of Bcl-2 (4IEH). Figure S10: 2D diagram of the interaction of HC030031 with Bcl-2 (4IEH). Figure S11: 2D and 3D diagrams of the interactions of compound ASP7663 with the crystal structure of Bax (1F16). Figure S12: 2D diagram of the interaction of HC030031 with Bax (1F16).
