# Supplementary material for: Pharmacologic Activation of TRPA1 Induces Multi-Target Anticancer Responses via Apoptotic and Mitochondrial Pathways
Source: Pharmaceuticals (Basel). 2026 Jul 13;19(7):1080. doi: 10.3390/ph19071080 (PMC13414800; doi:10.3390/ph19071080)
Supplement: Supplementary file 1 [file pharmaceuticals-19-01080-s001.zip › pharmaceuticals-4397721-supplementary.pdf]

Pharmacologic Activation of TRPA1 Induces Multitarget Anticancer Responses via Apoptotic and Mitochondrial Pathways

Running Head

Anti-Cancer Activities of ASP7663

Murat ÇAKIR[1*], Ali AYDIN[2], Burçin TÜRKMENOĞLU[3] & Mücahit SEÇME[4]

[1]Yozgat Bozok University, Faculty of Medicine, Department of Physiology, Yozgat, 66200, Türkiye
[2]Yozgat Bozok University, Faculty of Medicine, Department of Basic Medical Science, Yozgat, 66200, Türkiye
[3]Erzincan Binali Yıldırım University, Faculty of Pharmacy, Department of Analytical Chemistry, Erzincan, 24002, Türkiye
[4]Ordu University, Faculty of Medicine, Department of Basic Medical Science, Ordu, 52200, Türkiye

*Corresponding Author
Yozgat Bozok University, Faculty of Medicine, Department of Physiology, Yozgat, 66200, Türkiye
Phone: 0(354)217 89 91
E-mail: murat.cakir@yobu.edu.tr

ORCID
Murat ÇAKIR https://orcid.org/0000-0002-2066-829X
Ali AYDIN https://orcid.org/0000-0002-9550-9111
Burçin TÜRKMENOĞLU https://orcid.org/0000-0002-5770-0847
Mücahit SEÇME https://orcid.org/0000-0002-2084-760X

Supplementary Information

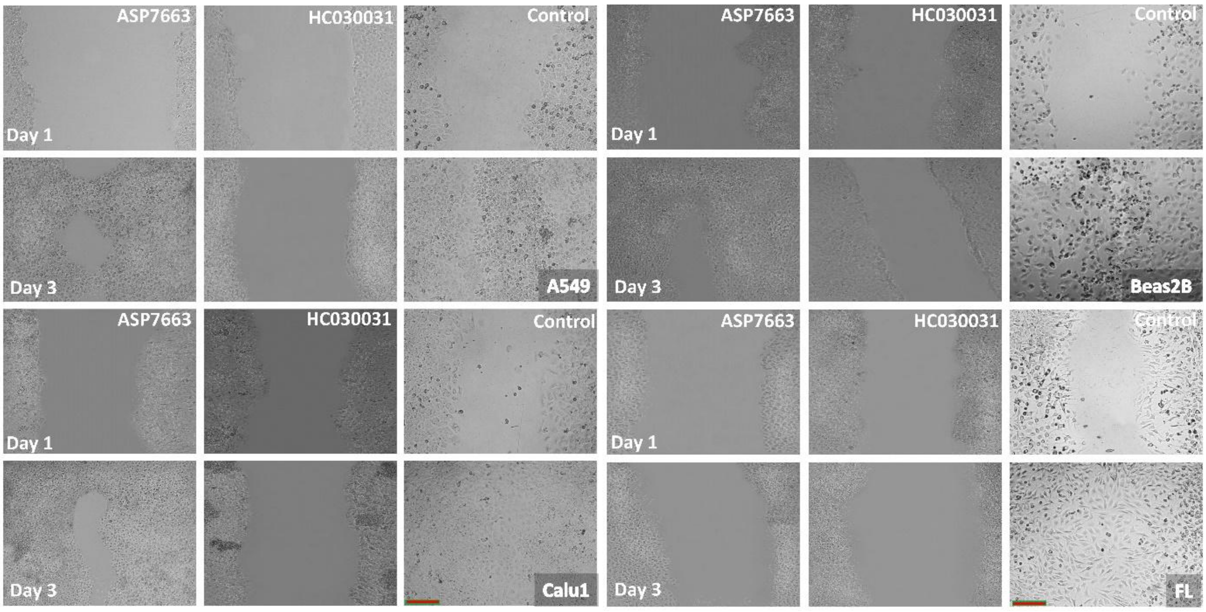

Supplemental Figure S1. Effects of ASP7663 and HC030031 molecules on cell migration in A549, Calu1, Beas2B, and FL cell lines. Tests were performed using cell lines showing exponential growth in log phase, and the incubation period was limited to 3 days.

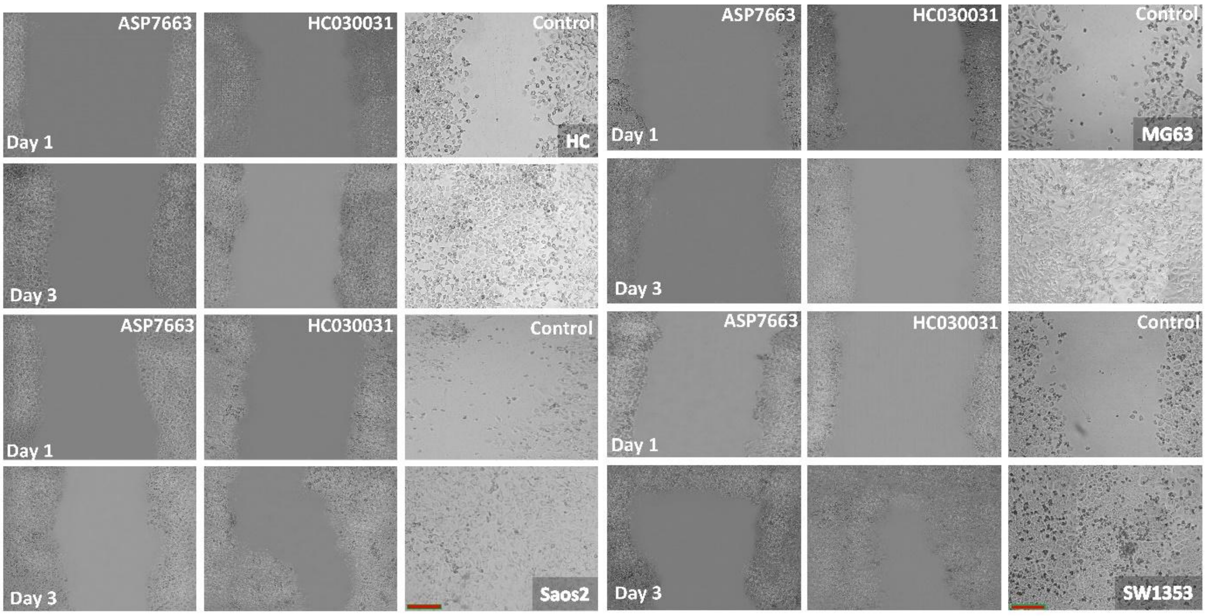

Supplemental Figure S2. Effects of ASP7663 and HC030031 molecules on cell migration in HC, Saos2, MG63, and SW1353 cell lines. Tests were performed using cell lines showing exponential growth in log phase, and the incubation period was limited to 3 days.

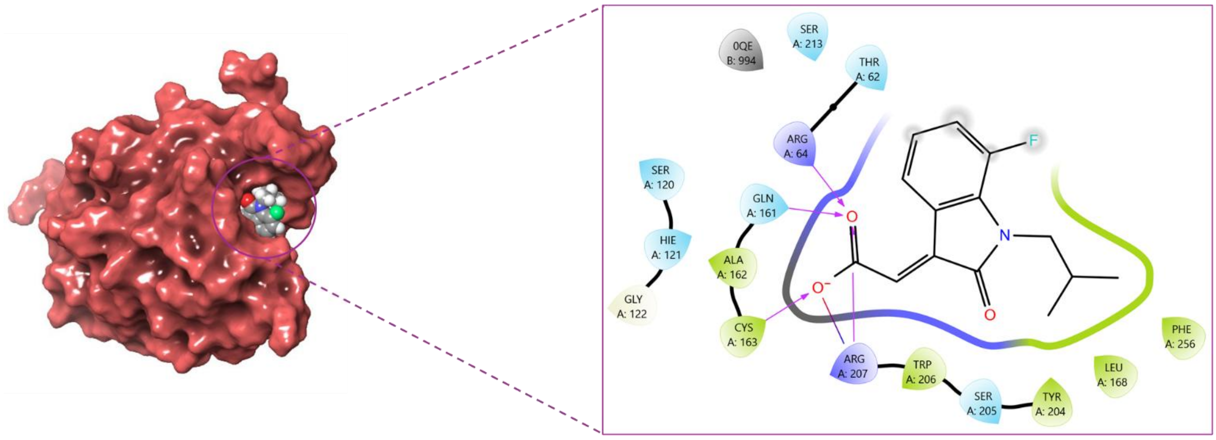

Supplemental Figure S3. 2D and 3D diagrams of the interactions of compound ASP7663 with the crystal structure of Caspase-3 (5IAG).

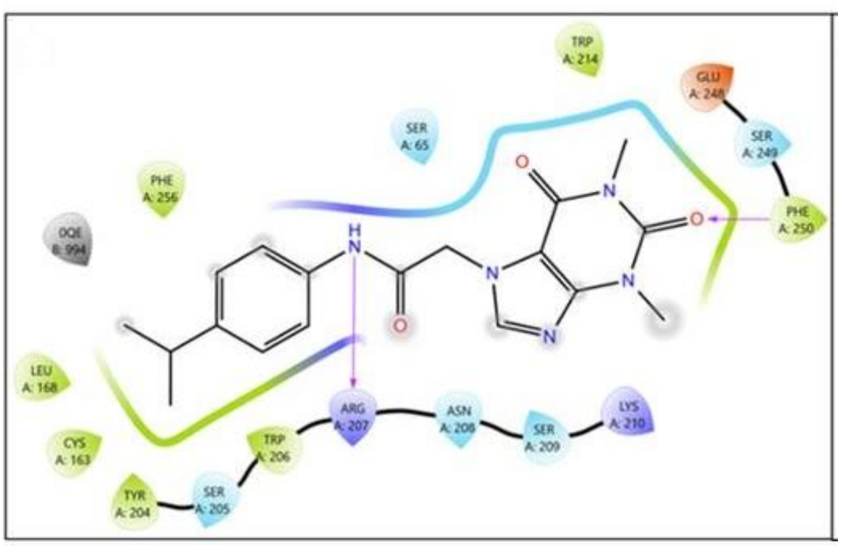

Supplemental Figure S4. 2D diagrams of the interactions of HC030031 with Caspase 3 (5IAG).

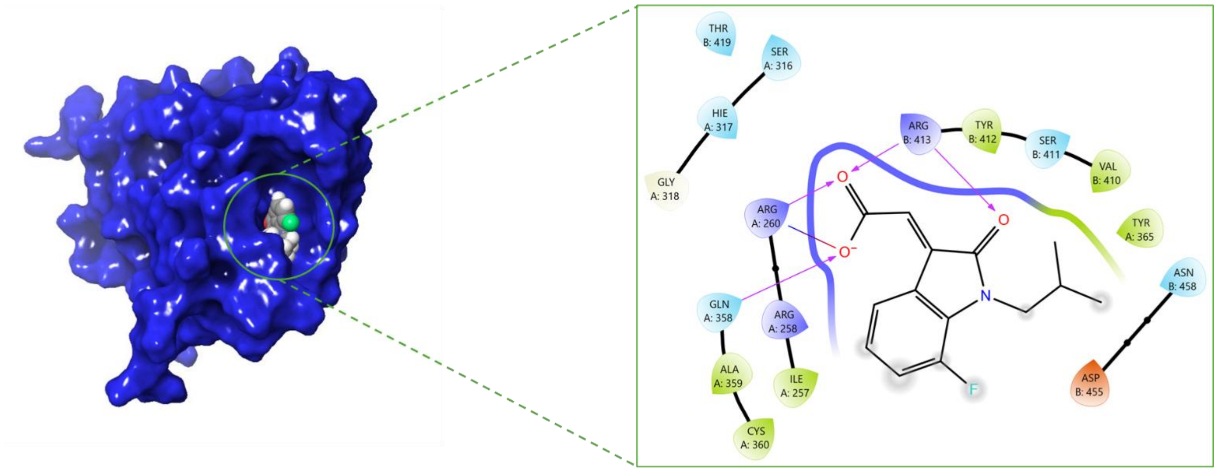

Supplemental Figure S5. 2D and 3D diagrams of the interactions of compound ASP7663 with the crystal structure of Caspase 8 (1QTN).

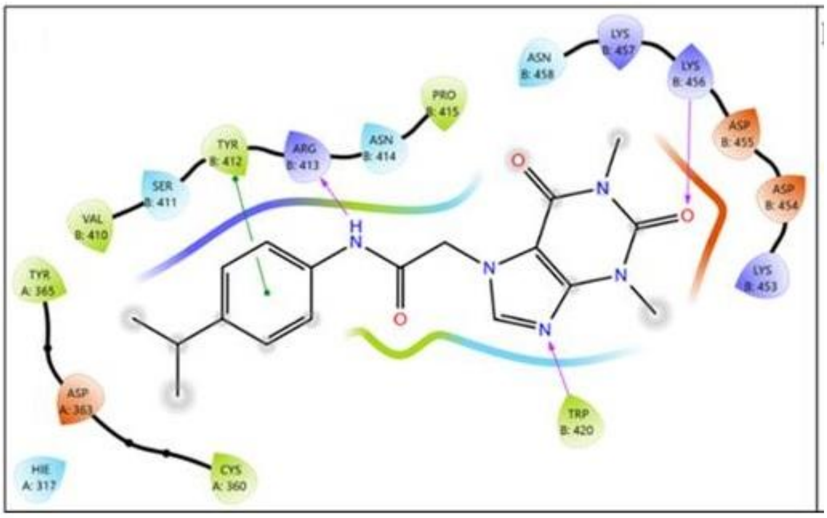

Supplemental Figure S6. 2D diagram of the interaction of HC030031 with Caspase-8 (1QTN).

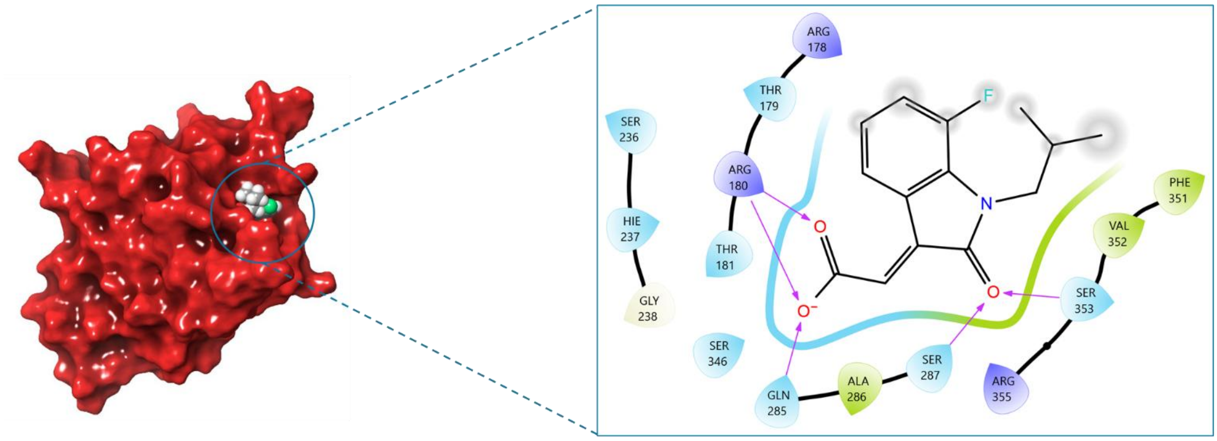

Supplemental Figure S7. 2D and 3D diagrams of the interactions of compound **ASP7663** with the crystal structure of Caspase 9 (2AR9).

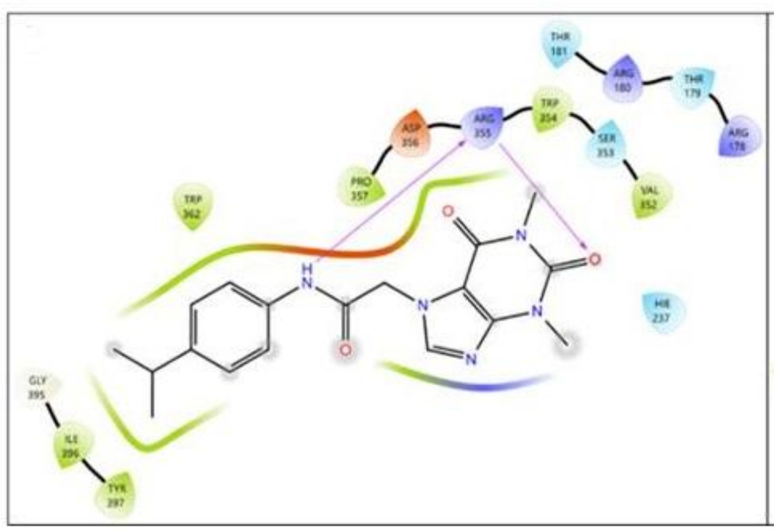

Supplemental Figure S8. 2D diagram of the interaction of HC030031 with Caspase-9 (2AR9).

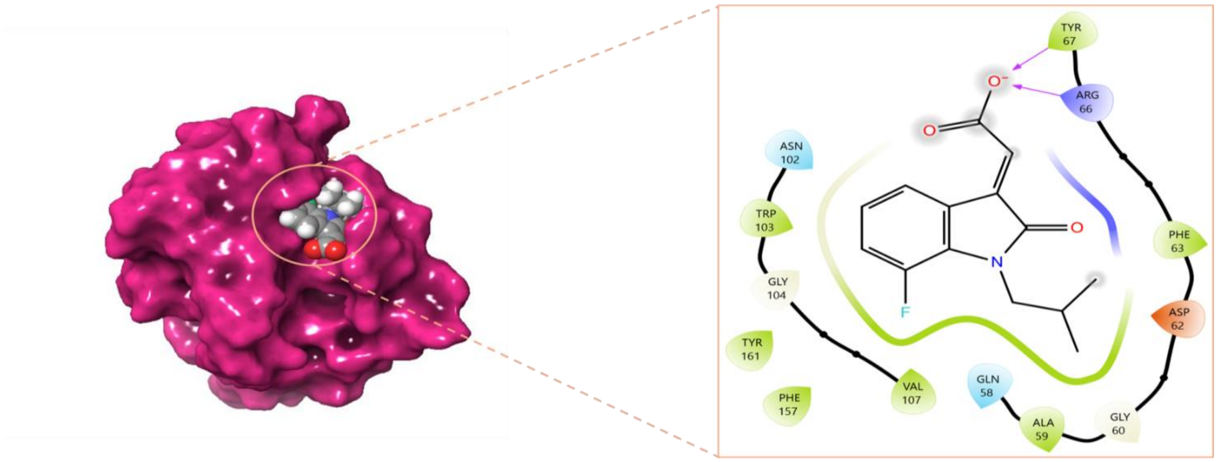

Supplemental Figure S9. 2D and 3D diagrams of the interactions of compound **ASP7663** with the crystal structure of Bcl-2 (4IEH).

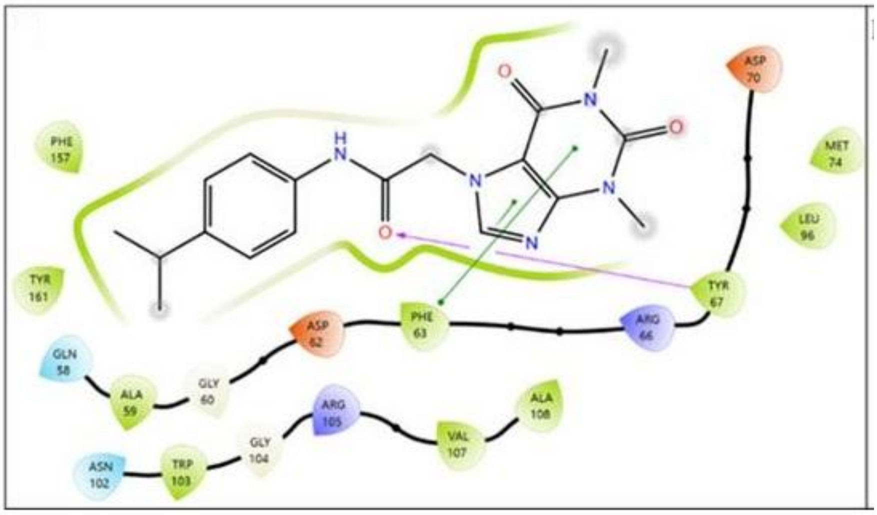

Supplemental Figure S10. 2D diagram of the interaction of HC030031 with Bcl-2 (4IEH).

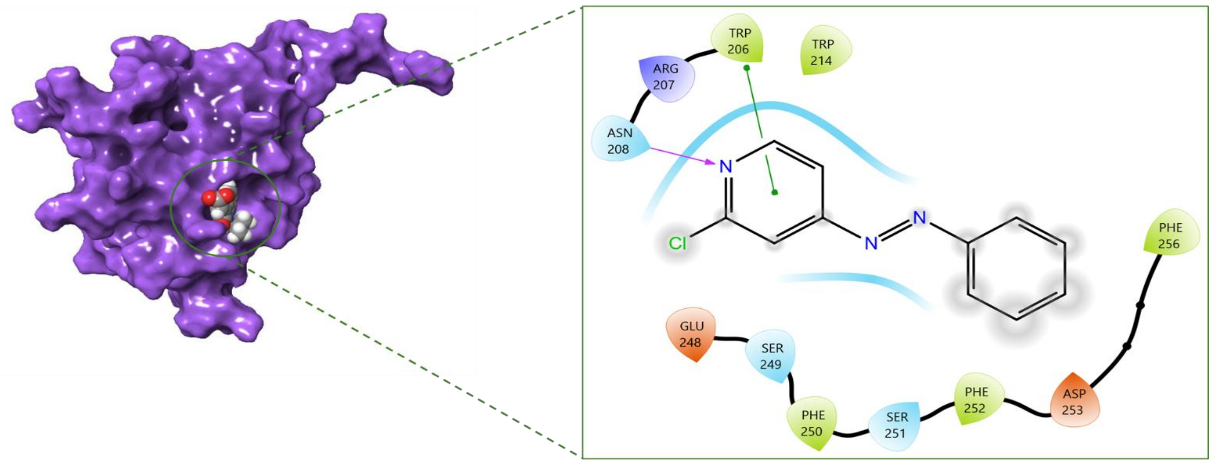

Supplemental Figure S11. 2D and 3D diagrams of the interactions of compound **ASP7663** with the crystal structure of Bax (1F16).

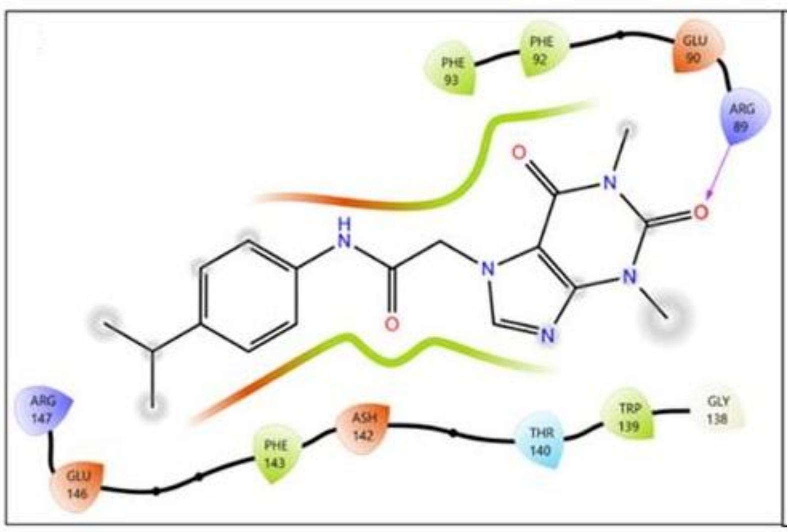

Supplemental Figure S12. 2D diagram of the interaction of HC030031 with Bax (1F16).
